# Mrs. Lucia T. S. Ingraham

**Published:** 1917-02

**Authors:** 


**Mrs. Lucia T. S. Ingraham,** widow of Dr. Henry D. Ingraham of Buffalo, died Jan. 3.

